# Comparison of ticagrelor and clopidogrel on platelet function and prognosis in unstable angina

**DOI:** 10.1007/s00228-022-03401-3

**Published:** 2022-10-17

**Authors:** Chun Li, Ming Liu, Weixiang Chen, Tingbo Jiang, Lin Ling

**Affiliations:** Department of Cardiovascular Medicine, First Affiliated Hospital of Soochow University, Suzhou, 215006 China

**Keywords:** Ticagrelor, Platelet aggregation rate, Cardiovascular prognosis, Bleeding, Unstable angina pectoris

## Abstract

**Purpose:**

This study aims to compare the effects of ticagrelor and clopidogrel on platelet function, cardiovascular prognosis, and bleeding in patients with unstable angina pectoris.

**Methods:**

Patients with unstable angina pectoris undergoing percutaneous coronary intervention (PCI) were enrolled (January 2018–December 2019). In total, 212 patients were treated with ticagrelor (90 mg twice daily) and 210 patients were treated with clopidogrel (75 mg once daily). Thromboelastography and light transmission aggregometry were used to measure the platelet aggregation rate (PAR). High-sensitivity troponin T (hs-TnT), pro-brain natriuretic peptide (NT-proBNP), high-sensitivity C-reactive protein (CRP), and heart-type fatty acid–binding protein (h-FABP) were measured to assess myocardial injury after PCI. Cardiovascular prognosis and bleeding events were evaluated in hospital and 12 months after discharge.

**Results:**

The PAR was significantly slower with ticagrelor (*P* < 0.001). hs-TnT, NT-proBNP, CRP, and h-FABP increased after compared with before PCI in both groups (*P* < 0.05). hs-TnT (*P* < 0.001) and h-FABP (*P* < 0.001) increased more significantly with clopidogrel. The in-hospital and 12-month major adverse cardiovascular event (MACE) rates were not significantly different between the two groups. The in-hospital total bleeding event rate was higher with ticagrelor (*P* < 0.05). Minor bleeding and total bleeding were more frequent at the 12-month follow-up in the ticagrelor group (*P* < 0.05).

**Conclusion:**

Ticagrelor was more effective in suppressing the PAR than clopidogrel and reduced PCI-induced myocardial injury in patients with unstable angina pectoris. However, it increased in-hospital and 12-month bleeding events and had no benefit on in-hospital and 12-month MACEs.

## Introduction

Acute coronary syndrome (ACS) is a leading cause of disability and death worldwide. Dual antiplatelet therapy (DAPT), including aspirin and P2Y12 inhibitors, is the current standard of treatment for ACS, especially in patients undergoing percutaneous coronary intervention (PCI). Clopidogrel is a classic P2Y12 receptor antagonist that is used worldwide [1–3]; however, it has a slow onset of action because it must first undergo hepatic bioactivation by cytochrome P450 2C19 (CYP2C19). Clopidogrel resistance in some patients due to *CYP2C19* loss of function results in a significant decrease in the concentration of clopidogrel in the blood and reduced drug effects.

Ticagrelor acts more rapidly and reversibly than clopidogrel, and current guidelines recommend ticagrelor for patients with ACS; however, studies in the real-world clinical setting have observed contradictory results, which suggest no superiority of ticagrelor over clopidogrel in specific populations [4–6].

In East Asian patients with unstable angina pectoris, the choice of ticagrelor or clopidogrel as DAPT, together with aspirin, is still controversial. To address this issue, this study retrospectively compared the effects of clopidogrel and ticagrelor on cardiovascular outcomes, platelet function, and bleeding events in Chinese patients with unstable angina pectoris who were successfully treated with PCI.

## Methods

### Study subjects

The data of patients who were diagnosed with unstable angina pectoris and who underwent successful PCI from January 2018 to December 2019 at the First Affiliated Hospital of Soochow University were retrospectively analyzed. The selection criteria for unstable angina pectoris were initial angina, worsening exertional angina, and resting angina with or without ischemia on electrocardiography. The exclusion criteria were (1) positive high-sensitivity troponin T (hs-TnT) at admission (> 5-times the upper limit); (2) thrombocytopenia (platelet count: < 50 × 10^9^/L) or a decreased hemoglobin concentration (Hb: < 10 g/dL) and no treatment with DAPT; and (3) PCI failure. The sample size calculation was performed in the pre-study; 136 patients in each group were needed for an *α* value of 0.05 and a power of 0.95. A total of 445 patients were enrolled, 422 of whom were included in this analysis and 23 of whom were lost to follow-up. Among the 422 patients, 212 were treated with ticagrelor (90 mg twice daily), while 210 were treated with clopidogrel (75 mg once daily). Patients in the clopidogrel group underwent *CYP2C19* testing. For patients with the slow metabolism genotype with the CYP2C9*2 or CYP2C9*3 homozygous mutant, we adjusted the medication to ticagrelor and excluded them from the study. All patients were also treated with aspirin (100 mg once daily). This study was approved by the Institutional Review Board of the First Affiliated Hospital of Soochow University, and all patients provided written informed consent before participation.

### Patient and public involvement

After patients were admitted to the hospital, we evaluated and selected patients who met the inclusion criteria, and we informed patients of the details of this study. All patients volunteered to participate in this study and provided written informed consent. During the study, patients were required to cooperate with the investigators for blood tests, follow-up, and observation of cardiovascular events. There was no additional cost to patients in this study. The timing, content, and possible risks and benefits of this study were fully described to patients.

### Clinical data collection

The basic clinical data of patients were recorded, including the general condition; comorbidities; tobacco and alcohol habits; bleeding history; routine blood test results; liver and kidney function; blood lipid, cardiac marker, and hs-TnT concentrations; echocardiography indicators; and medications. Elective PCI was performed, and coronary artery conditions, including the number of diseased vessels, thrombolysis in myocardial infarction flow grade, and the number of stents and balloons, were recorded.

### Determination of platelet function

All patients underwent blood tests on days 0, 3, and 30 of treatment. Platelet function induced by adenosine diphosphate (ADP) and arachidonic acid (AA) was measured by thromboelastography (TEG) and light transmission aggregometry (LTA). The TEG device manufactured by Haemoscope Company (USA) and the platelet function analyzer manufactured by Nanjing Xierjian Medical Instrument Co. Ltd. (China) were used.

### Detection of myocardial injury after PCI

Blood samples before and 24 h after PCI were analyzed to determine the concentrations of hs-TnT, N-terminal pro-brain natriuretic peptide (NT-proBNP), high-sensitivity C-reactive protein (hs-CRP), and heart-type fatty acid–binding protein (h-FABP). Chemiluminescence and enzyme-linked immunosorbent assays were used for detection, and the detection kit was provided by Nanjing FCMCS Biotechnology Co. Ltd. (China).

### Cardiovascular prognosis and bleeding events

In-hospital and 12-month cardiovascular prognosis and bleeding events were assessed. Cardiovascular endpoints included all-cause mortality, myocardial infarction (MI), target vessel revascularization (TVR), stent thrombosis, stroke, transient ischemic attack (TIA), and overall major adverse cardiovascular events (MACEs). The Bleeding Academic Research Consortium criteria were used to define the degree of bleeding. Minor bleeding was defined as skin bruising, subcutaneous ecchymosis, nosebleed, or bleeding gums, while major bleeding was defined as fatal bleeding, significant bleeding requiring blood transfusion, gastrointestinal bleeding, or intracranial hemorrhage with a decrease in Hb concentration of ≥ 3 g/dL.

### Statistical analysis

SPSS 22.0 software was used for statistical analysis. Count data are expressed as rates, which were compared using the chi-square test. Measurement data are expressed as mean ± standard deviation (x ± s). The data were tested for normality prior to statistical analysis. If the data were normally distributed, we used the unpaired parametric *t*-test with Welch’s correction. If the normality test indicated that the data did not meet the criteria for parametric testing, we performed the Kruskal–Wallis test followed by the Mann–Whitney *U* test as a post hoc test. A *P* value of < 0.05 was considered statistically significant.

## Results

### Patients’ clinical characteristics

Patients’ basic clinical characteristics, including blood test results, medications, coronary artery procedures, and echocardiography indices, are listed in Table 1. No significant differences in these clinical indices were observed between the clopidogrel and ticagrelor groups.

**Table 1:** Patients’ baseline characteristics

Index	Clopidogrel (*n* = 210)	Ticagrelor (*n* = 212)	*P* value
Clinical data			
Male (%)	70% (147)	76.41% (162)	0.137
Age (years)	62.86 ± 15.06	64.76 ± 14.53	0.186
Body mass index (kg/m^2^) Hypertension (%)	23.92 ± 2.37 61.9% (130)	24.53 ± 4.53 66% (140)	0.079 0.377
Diabetes mellitus (%)	38% (80)	41.98% (89)	0.415
Smoker (%)	48% (101)	52.35% (111)	0.381
Drinker (%) Previous GI bleeding (%) Peptic ulcer (%) Previous CVD (%)	20% (42) 1.42% (3) 9.52% (20) 9% (19)	25.94% (55) 0.94% (2) 6.13% (13) 12.26% (26)	0.147 0.645 0.194 0.284
Hemoglobin (g/L)	130.14 ± 21.62	129.18 ± 29.12	0.701
Triglyceride (mmol/L)	2.74 ± 1.03	2.58 ± 1.04	0.113
Cholesterol (mmol/L)	5.32 ± 1.57	5.11 ± 1.29	0.134
LDL-C (mmol/L) HbA1c (%)	3.13 ± 1.38 5.52 ± 0.89	2.90 ± 1.24 5.41 ± 0.88	0.072 0.202
Creatinine (µmol/L)	89.37 ± 35.26	87.91 ± 30.57	0.649
BUN (µmol/L)	6.75 ± 2.21	6.56 ± 2.13	0.369
ALT (U/L)	32.18 ± 14.61	30.17 ± 12.56	0.131
CK (U/L)	279.92 ± 267.05	238.61 ± 183.14	0.065
hs-TnT (µg/L)	49.19 ± 28.27	54.13 ± 26.22	0.063
Medications			
Aspirin	100%	100%	1
ACEI/ARB Beta-blockers	74.28% (156) 49.52% (105)	68.39% (145) 40.09% (95)	0.181 0.286
Statins			
PPI Coronary procedure Radial	100% 100% 100%	100% 100% 100%	1 1 1
No. of diseased arteries	1.86 ± 0.49	1.75 ± 0.79	0.086
No. of stents			
No. of balloons TIMI flow grade Echocardiography indices LVEF (%) LVEDd (mm) LVESd (mm) LAD (mm)	1.15 ± 0.39 2.1 ± 0.32 2.88 ± 0.38 51.84 ± 8.25 53.34 ± 6.82 42.67 ± 6.17 42.15 ± 4.93	1.22 ± 0.46 2.04 ± 0.60 2.86 ± 0.45 53.13 ± 9.21 52.18 ± 6.92 41.65 ± 6.37 42.94 ± 5.08	0.092 0.200 0.622 0.131 0.084 0.096 0.106

*GI* gastrointestinal, *CVD* cardiovascular disease, *LDL-C* low-density lipoprotein cholesterol, *HbA1c* glycated hemoglobin, *BUN* blood urea nitrogen, *ALT* alanine aminotransferase, *CK* creatine kinase, *hs-TnT* high-sensitivity troponin T, *ACEI/ARB* angiotensin-converting enzyme inhibitor/angiotensin receptor antagonist, *PPI* proton pump inhibitor, *LVEF* left ventricular ejection fraction, *LVEDd* left ventricular end-diastolic diameter, *LVESd* left ventricular end-systolic diameter, *LAD* left atrial diameter

### Comparison of platelet aggregation rate (PAR) between the clopidogrel and ticagrelor groups

**Fig. 1:**
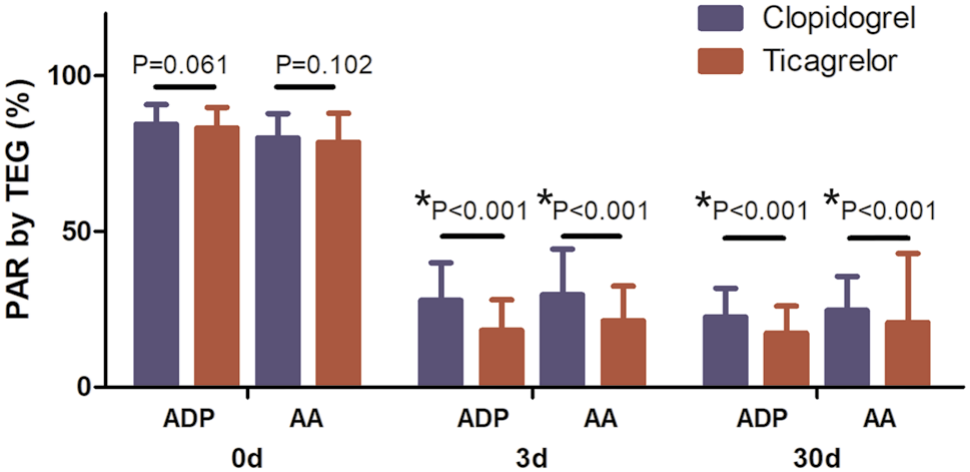
PAR assessed by TEG at different time points. On days 3 and 30, the PAR in the ticagrelor group was significantly slower than in the clopidogrel group (*P* < 0.001). Data are presented as mean ± standard deviation. PAR, platelet aggregation rate; TEG, thromboelastography; ADP, adenosine diphosphate; AA, arachidonic acid

**Fig. 2:**
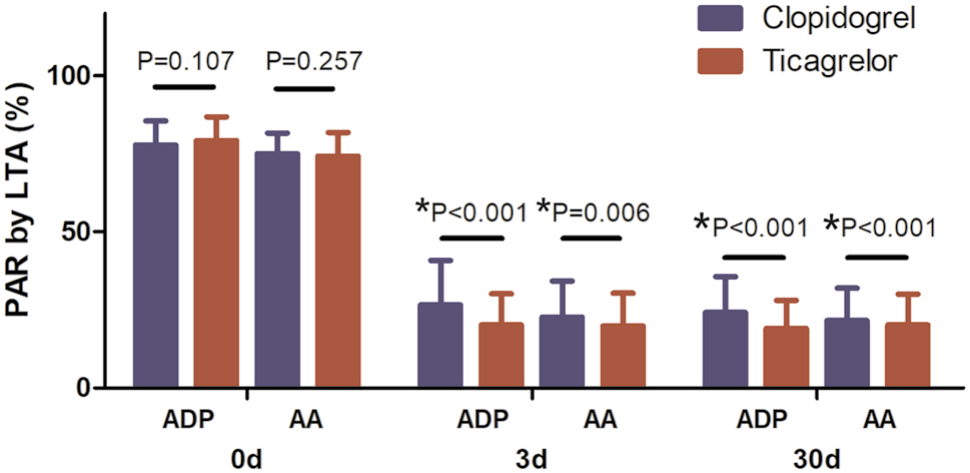
PAR assessed by LTA at different time points. On days 3 and 30, the PAR in the ticagrelor group was significantly slower than in the clopidogrel group (*P* < 0.001). Data are presented as mean ± standard deviation. PAR, platelet aggregation rate; LTA, light transmission aggregometry; ADP, adenosine diphosphate; AA, arachidonic acid

**Fig. 3:**
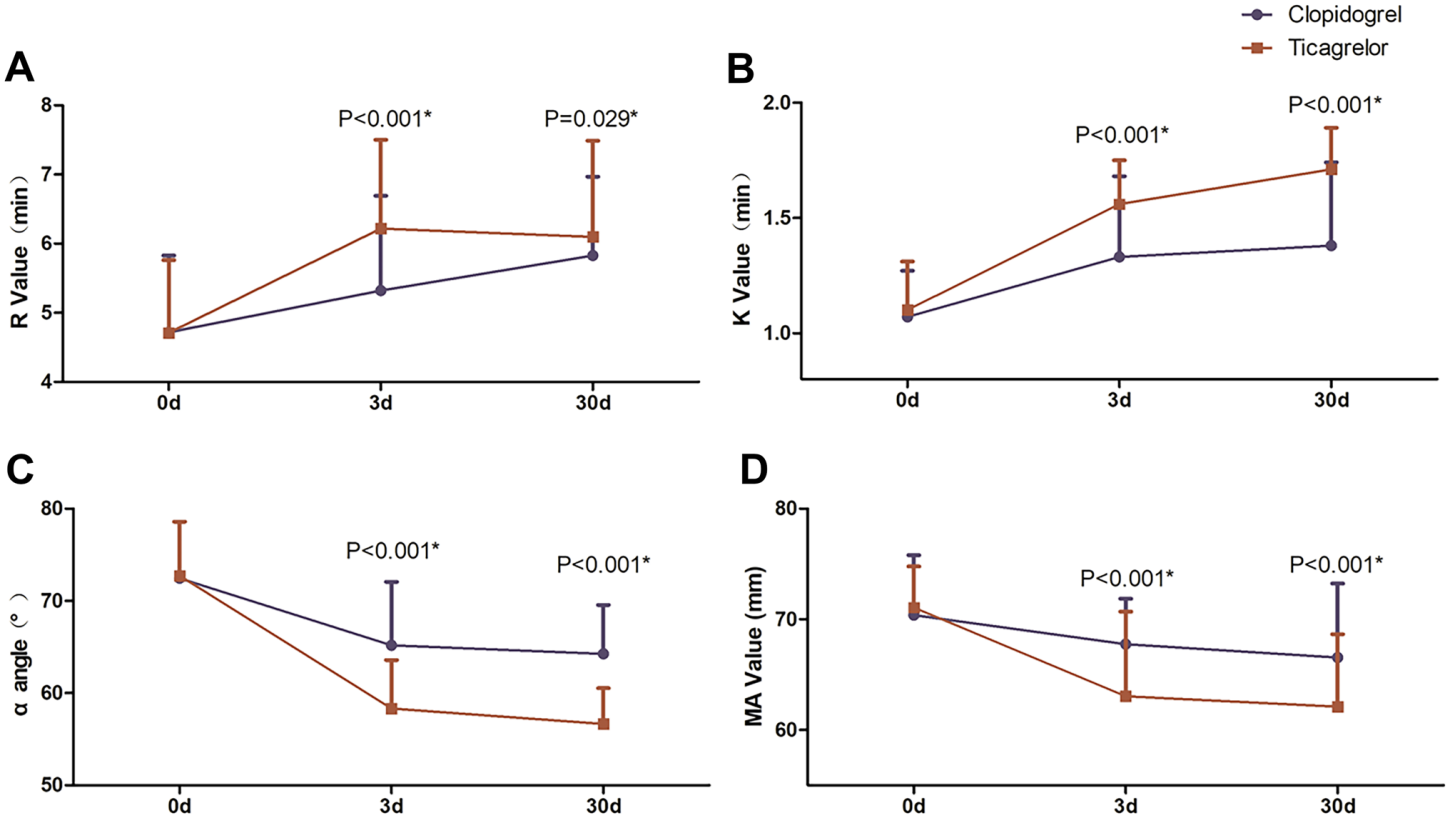
TEG showed that the *R* and *K* values were prolonged and the *α* angle and MA value were lower in the ticagrelor group than in the clopidogrel group. Data are presented as mean ± standard deviation. TEG, thromboelastography; MA, mean maximal amplitude

Compared with the clopidogrel group, the PAR in the ticagrelor group induced by ADP and AA decreased on days 3 and 30, and the difference between the two groups was statistically significant. Both TEG and LTA showed the same trend (*P* < 0.001; Figs. 1 and 2). Ticagrelor was more effective in platelet suppression than clopidogrel. TEG showed that the *R* and *K* values were prolonged, and the *α* angle and mean maximal amplitude (MA) were significantly decreased in the ticagrelor group compared with the clopidogrel group (Fig. 3) (*P* < 0.05). The data are presented in detail in Table 2.

**Table 2:** Comparison of PAR between the clopidogrel and ticagrelor groups

Index	Clopidogrel (*n* = 210)	Ticagrelor (*n* = 212)	*P* value
ADP-PAR by TEG (%)			
Day 0 Day 3	84.44 ± 6.34 27.86 ± 12.05	83.25 ± 6.58 18.23 ± 9.86	0.059 < 0.001*
Day 30	22.52 ± 9.21	17.38 ± 8.71	< 0.001*
AA-PAR by TEG (%)			
Day 0 Day 3 Day 30	80.06 ± 7.79 29.64 ± 14.72 24.63 ± 10.88	78.66 ± 9.31 21.30 ± 11.17 20.73 ± 22.24	0.095 < 0.001* < 0.001*
ADP-PAR by LTA (%)			
Day 0 Day 3 Day 30	77.89 ± 7.64 26.62 ± 14.37 24.24 ± 11.53	79.21 ± 7.64 20.28 ± 9.93 19.18 ± 9.01	0.077 < 0.001* < 0.001*
AA-PAR by LTA (%)			
Day 0 Day 3 Day 30	75.01 ± 6.69 22.76 ± 11.55 21.69 ± 10.31	74.25 ± 7.61 19.92 ± 10.49 20.25 ± 9.94	0.276 0.009* < 0.001*
*R* value (min)			
Day 0 Day 3	4.72 ± 1.11 5.32 ± 1.37	4.71 ± 1.05 6.22 ± 1.28	0.924 < 0.001*
Day 30	5.83 ± 1.14	6.10 ± 1.39	0.029*
*K value (min)*			
Day 0 Day 3 Day 30	1.07 ± 0.20 1.33±0.35 1.38 ± 0.36	1.10 ± 0.21 1.56 ± 0.19 1.71 ± 0.18	0.134 < 0.001* < 0.001*
*α* angle (°)			
Day 0 Day 3 Day 30	72.47 ± 6.11 65.19 ± 6.88 64.26 ± 5.31	72.74 ± 5.86 58.33 ± 5.26 56.67 ± 3.87	< 0.001* 0.643 < 0.001*
MA value (mm)			
Day 0 Day 3 Day 30	70.39 ± 5.42 67.76 ± 4.11 66.56 ± 6.67	71.08 ± 3.71 63.06 ± 7.64 62.11 ± 6.54	0.128 < 0.001* < 0.001*

^*^P < 0.001 ticagrelor vs. clopidogrel. Data are presented as mean ± standard deviation

*PAR* platelet aggregation rate, *ADP* adenosine diphosphate, *TEG* thromboelastography, *AA* arachidonic acid, *LTA* light transmission aggregometry, *MA* mean maximal amplitude

### PCI-related myocardial injury assessment

**Fig. 4:**
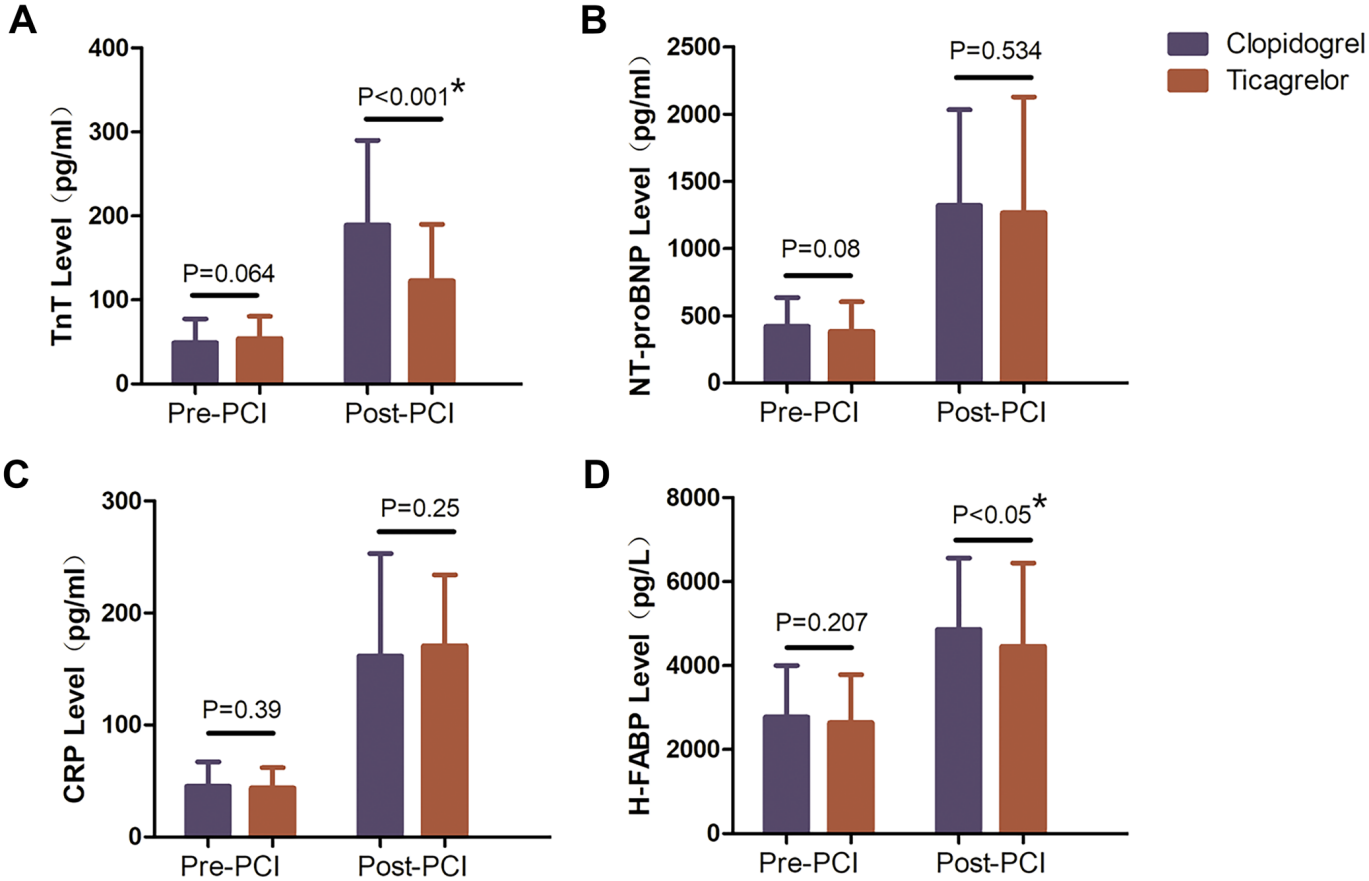
Blood hs-TnT, NT-proBNP, CRP, and h-FABP concentrations before and after PCI in the clopidogrel and ticagrelor groups. hs-TnT, NT-proBNP, CRP, and h-FABP concentrations were higher after PCI than before PCI (*P* < 0.05 for all). hs-TnT (*P* < 0.001) and h-FABP (*P* < 0.001) increased more significantly after PCI in the clopidogrel group than in the ticagrelor group. Data are presented as mean ± standard deviation. hs-TnT, high-sensitivity troponin T; NT-proBNP, N-terminal pro-brain natriuretic peptide; CRP, C-reactive protein; h-FABP, heart-type fatty acid–binding protein; PCI, percutaneous coronary intervention

Before PCI, there were no significant differences between the two groups in the blood concentrations of hs-TnT (49.19 ± 28.27 µg/L vs. 54.12 ± 26.22 µg/L; *P* = 0.064), NT-proBNP (419.16 ± 214.82 pg/mL vs. 382.02 ± 221.62 pg/mL; *P* = 0.081), CRP (45.34 ± 21.69 pg/mL vs. 43.79 ± 18.11 pg/mL; *P* = 0.426), and h-FABP (2777.01 ± 1225.65 pg/L vs. 2642.62 ± 1144.05 pg/L; *P* = 0.245). hs-TnT (clopidogrel: 189.08 ± 101.05 µg/L vs. 49.19 ± 28.27 µg/L; ticagrelor: 122.84 ± 67.46 µg/L vs. 54.12 ± 26.22 µg/L), NT-proBNP (clopidogrel: 1320.50 ± 711.25 pg/mL vs. 419.16 ± 214.82 pg/mL; ticagrelor: 1265.75 ± 863.16 pg/mL vs. 382.02 ± 221.62 pg/mL), CRP (clopidogrel: 161.73 ± 91.46 pg/mL vs. 45.34 ± 21.69 pg/mL; ticagrelor: 170.97 ± 63.26 pg/mL vs. 43.79 ± 18.11 pg/mL), and h-FABP (clopidogrel: 4860.63 ± 1701.22 pg/L vs. 2777.01 ± 1225.65 pg/L; ticagrelor: 4465.03 ± 1980.89 pg/L vs. 2642.62 ± 1144.05 pg/L) were higher after PCI than before PCI (*P* < 0.001 for all). hs-TnT (clopidogrel: 189.08 ± 101.05 µg/L vs. ticagrelor: 122.84 ± 67.46 µg/L; *P* < 0.001) and h-FABP (clopidogrel: 4860.63 ± 1701.22 pg/L vs. ticagrelor: 4465.03 ± 1980.89 pg/L; *P* < 0.05) were significantly higher in the clopidogrel group than in the ticagrelor group (Fig. 4).

### Cardiovascular prognosis and bleeding events

**Fig. 5:**
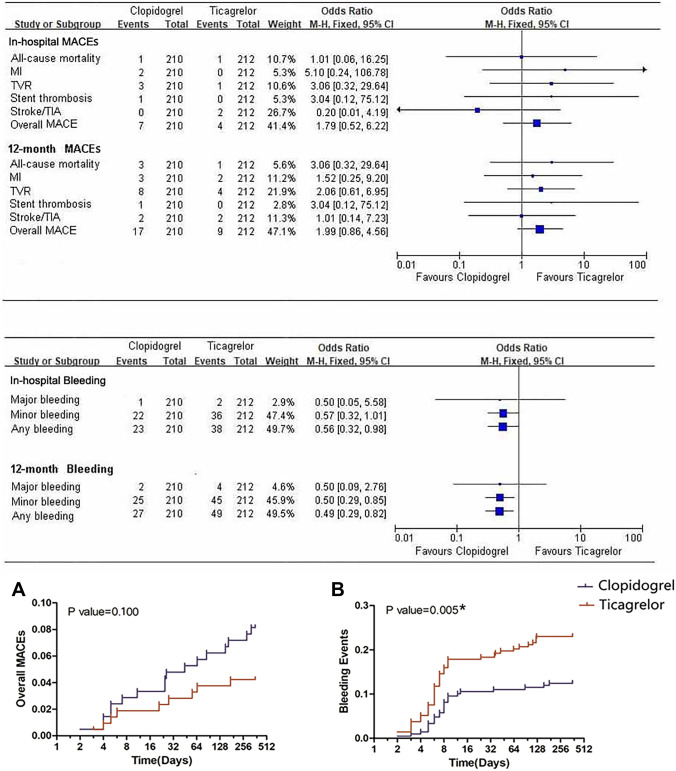
Kaplan–Meier curves for overall MACEs and bleeding outcomes with ticagrelor versus clopidogrel. MACEs showed no significant difference between the two groups, and in-hospital and 12-month bleeding events were significantly more frequent in the ticagrelor group than in the clopidogrel group. MACEs, major adverse cardiovascular events; MI, myocardial infarction; TVR, target vessel revascularization; TIA, transient ischemic attack; CI, confidence interval

In-hospital and 12-month (post-discharge) cardiovascular prognosis was evaluated, including all-cause mortality, MI, TVR, stent thrombosis, stroke, TIA, and overall MACEs. In-hospital and 12-month MACEs were not significantly different between the clopidogrel and ticagrelor groups. In-hospital total bleeding events were more frequent in the ticagrelor group than in the clopidogrel group. Both minor bleeding and total bleeding events were significantly more frequent at the 12-month follow-up in the ticagrelor group than in the clopidogrel group (*P* < 0.05). The Kaplan–Meier curves for overall MACEs and bleeding outcomes ae shown in Fig. 5. The detailed results are shown in Table 3.

**Table 3:** In-hospital and 12-month cardiovascular prognosis in the clopidogrel and ticagrelor groups

Endpoints	Clopidogrel (*n* = 210)	Ticagrelor (*n* = 212)	*P* value
**In-hospital MACEs (*n*)**			
All-cause mortality	1	1	0.995
MI	2	0	0.154
TVR	3	1	0.31
Stent thrombosis	1	0	0.314
Stroke/TIA	0	2	0.158
Overall MACEs	7	4	0.351
		1	
**MACEs at 12-month follow-up (*n*)**		2	0.557
All-cause mortality	3	4	0.645
MI	3	0	0.512
TVR	8	2	0.314
Stent thrombosis	1	9	0.992
Stroke/TIA	2		0.1
Overall MACEs	17	2	0.568
		36	0.052
**In-hospital bleeding (*n*)**	1	38	0.042*
Major bleeding	22		0.418
Minor bleeding	23	4	0.01*
Any bleeding		45	0.005*
	2	49	
**Bleeding at 12-month follow-up (*n*)**	25		
Major bleeding	27		
Minor bleeding			
Any bleeding			

*MACEs* major adverse cardiovascular events, *MI* myocardial infarction, *TVR* target vessel revascularization, *TIA* transient ischemic attack

^*^P < 0.05 ticagrelor vs. clopidogrel

## Discussion

Antiplatelet therapy is the standard treatment for ACS [7], and clopidogrel is widely used as one of the DAPT therapies. However, clopidogrel has certain limitations. As a prodrug, clopidogrel needs to undergo hepatic metabolism by CYP2C19 after oral administration, which slows its onset of action. It cannot achieve rapid platelet inhibition in patients with acute MI who require emergency surgery. Moreover, clopidogrel metabolism varies between patients. Moreover, due to individual genetic variation, some patients demonstrate metabolic resistance to clopidogrel, preventing its antiplatelet effect. Ischemic events are more frequent in these patients. In addition, clopidogrel causes irreversible platelet aggregation, which leads to a longer platelet function recovery time after clopidogrel discontinuation, and it cannot be used in patients who require rapid reversal of the antiplatelet effect. In comparison, ticagrelor is a reversible P2Y12 receptor antagonist that does not require hepatic metabolism for activation; thus, it acts more rapidly than clopidogrel. Moreover, individual genetic variation does not affect the efficacy of ticagrelor, and ticagrelor can quickly inhibit platelet aggregation [8–11].

The PLATO trial compared ticagrelor with clopidogrel in high-risk ACS patients. Ticagrelor decreased the incidence of the primary composite endpoint of cardiovascular death, MI, and stroke, but there was no significant difference in overall severe bleeding. Based on the PLATO trial, current international guidelines recommend the use of ticagrelor prior to clopidogrel in patients with ACS [12–14]. In recent years, the use of ticagrelor in patients with ACS has rapidly increased in Asian countries, including in patients with unstable angina pectoris and MI; however, the clinical outcomes and bleeding risk of ticagrelor in this population are unknown. Few studies have examined platelet aggregation and PCI-related myocardial injury in patients with unstable angina pectoris, which we examined in this study.

The results of this study showed that compared with clopidogrel, the ADP- and AA-induced PARs decreased significantly after treatment with ticagrelor, with prolonged *R* and *K* values and a decreased *α* angle and MA value. This suggests that ticagrelor is more effective in inhibiting platelet aggregation and activation than clopidogrel. In the ONSET/OFFSET study, the PAR was 60% at 30 min after 180-mg ticagrelor loading dose and 10% at 2–4 h after administration, with a corresponding platelet inhibition rate of 90%. In our study, two time points (3 days and 30 days) were selected to determine the PAR after treatment with ticagrelor, which demonstrated a steady-state drug concentration. The results showed that the ADP-induced PAR was 18.23% ± 9.86% at 3 days and 17.38% ± 8.71% at 30 days in the ticagrelor group, as assessed by TEG. The AA-induced PAR was 21.30% ± 11.17% at 3 days and 20.73% ± 22.24% at 30 days. LTA showed comparable results to TEG. Specifically, the ADP-induced PAR in the ticagrelor group was 20.28% ± 9.93% at 3 days and 19.18% ± 9.01% at 30 days in the ticagrelor group. The AA-induced PAR was 19.92% ± 10.49% at 3 days and 20.25% ± 9.94% at 30 days in the ticagrelor group. Compared with the clopidogrel group, the AA- and ADP-induced PARs in the ticagrelor group were significantly lower. These results suggest that ticagrelor can continuously and steadily inhibit platelet activation more effectively than clopidogrel, which is consistent with the ONSET/OFFSET study [15].

AA-induced platelet aggregation depends on the activity of cyclooxygenase-1 (COX1). Both ticagrelor and clopidogrel inhibit the P2Y12 receptor, which is downstream of the COX-1 pathway. Our results indicate that ticagrelor may be superior to clopidogrel in its ability to inhibit ADP-induced and AA-induced platelet aggregation.

PCI-related myocardial injury is very common because it can cause further damage to the plaque or thrombus on the inner wall of the coronary artery, increasing the risk of recurrent myocardial ischemia and myocardial injury after PCI. A recent study showed that compared with clopidogrel, loading-dose pretreatment with ticagrelor can significantly reduce the incidence of PCI-related periprocedural MI in Asian patients with ACS undergoing elective PCI. A multivariate analysis found that the use of ticagrelor was negatively correlated with PCI-related periprocedural MI, indicating that ticagrelor treatment is an independent protective predictor of periprocedural MI [16]. PCI-related MI is defined as an elevation in cardiac hs-TnT concentration > 5-times the 99th percentile upper reference limit. hs-TnT is recognized as a specific indicator of myocardial injury. In addition, h-FABP leaks from damaged cardiomyocytes. As such, both hs-TnT and h-FABP are sensitive indicators of myocardial injury. Previous studies have reported that the concentration of h-FABP in peripheral blood in patients with MI is related to coronary artery disease severity and can be used to assess the area of MI and cardiovascular prognosis in patients with acute ST-segment elevation MI (STEMI) [17]. The results of our study show that the increase in hs-TnT and h-FABP after PCI in the ticagrelor group was significantly smaller than in the clopidogrel group. This suggests that ticagrelor could protect against myocardial damage.

Patients in the ticagrelor group tended to present with fewer cardiovascular events, but in-hospital MACEs and 12-month MACEs were not significantly different between the two groups. In-hospital and 12-month bleeding events were more common in the ticagrelor group. This agrees with several recent studies, which reached the same conclusions. Clopidogrel has been proven as noninferior to ticagrelor in cardiovascular outcomes, with fewer bleeding events in several populations, including older patients aged > 70 years with ACS, patients with STEMI, patients in the intensive care unit with ACS, non-STEMI patients with ACS, patients with ACS undergoing PCI, and older patients with non-STEMI with ACS and combined use of novel anticoagulants, amongst others [18–23].

The ISAR-REACT-5 trial showed that prasugrel reduced the rate of death, MI, and stroke at 1 year compared with ticagrelor among patients with ACS undergoing PCI, with no significant difference in bleeding. These results are similar to ours [24]. An increasing amount of real-world evidence has shown that ticagrelor is not superior to clopidogrel or prasugrel in terms of cardiovascular benefits, and it may be associated with a higher risk of bleeding. Therefore, based on the results of our study, we believe that clopidogrel should be preferentially used as an antiplatelet drug in patients with unstable angina undergoing elective PCI in Asia, especially those with a high bleeding risk.

In the study of Xu et al., in elderly Chinese patients with coronary artery disease, ticagrelor was associated with a lower incidence of major cardiovascular adverse events at 12 months than clopidogrel, while bleeding events were not significantly increased. However, the two groups differed in terms of the proportion of patients who underwent coronary intervention. Specifically, a higher proportion of patients underwent coronary intervention in the ticagrelor group. In our study, the proportion of patients who underwent coronary intervention was the same in both groups. The high proportion of patients who underwent intervention suggests that patients may achieve better revascularization, thus leading to a better cardiovascular outcome [25]. Our study has some limitations that should be noted. The sample size was relatively small, and the follow-up time was only 12 months. Future large-sample studies with longer follow-up times should be conducted in the future to validate our findings.

In conclusion, our study showed that ticagrelor was more effective in suppressing platelet aggregation than clopidogrel. Moreover, ticagrelor may reduce myocardial injury as a result of PCI in patients with unstable angina pectoris. However, ticagrelor showed no advantages over clopidogrel in reducing in-hospital and 12-month MACEs, and it increased in-hospital and 12-month bleeding events.

## Data Availability

Not applicable.

## Abbreviations

ACS: Acute coronary syndrome
PCI: Percutaneous coronary intervention
CYP2C19: Cytochrome P450 2C19
DAPT: Dual antiplatelet therapy
hs-TnT: High-sensitivity troponin T
PAR: Platelet aggregation rate
ADP: Adenosine diphosphate
AA: Arachidonic acid
TEG: Thromboelastography
LTA: Light transmission aggregometry
MA: Mean maximal amplitude
NT-proBNP: N-Terminal pro-brain natriuretic peptide
hs-CRP: High-sensitivity C-reactive protein
h-FABP: Heart-type fatty acid–binding protein
MI: Myocardial infarction
TVR: Target vessel revascularization
MACEs: Major adverse cardiovascular events
GI: Gastrointestinal
CVD: Cardiovascular disease
LDL-C: Low-density lipoprotein cholesterol
HbA1c: Glycated hemoglobin
BUN: Blood urea nitrogen
ALT: Alanine aminotransferase
CK: Creatine kinase
ACEI/ARB: Angiotensin-converting enzyme inhibitor/angiotensin receptor antagonist
PPI: Proton pump inhibitor
LVEF: Left ventricular ejection fraction
LVEDd: Left ventricular end-diastolic diameter
LVESd: Left ventricular end-systolic diameter
LAD: Left atrial diameter
TIA: Transient ischemic attack
COX1: Cyclooxygenase-1
